# Vitamin D-Related Genetic Variations and Nonalcoholic Fatty Liver Disease: A Systematic Review

**DOI:** 10.3390/ijms23169122

**Published:** 2022-08-14

**Authors:** Aunchalee Jaroenlapnopparat, Pichatorn Suppakitjanusant, Ben Ponvilawan, Nipith Charoenngam

**Affiliations:** 1 Department of Medicine, Mount Auburn Hospital, Beth Israel Lahey Health, Cambridge, MA 02138, USA; 2 Department of Medicine, Harvard Medical School, Boston, MA 02115, USA; 3 Division of Endocrinology, Metabolism, and Lipids, Emory University School of Medicine, Atlanta, GA 30322, USA; 4 Department of Internal Medicine, University of Missouri—Kansas City School of Medicine, Kansas City, MO 64108, USA; 5 Department of Medicine, Faculty of Medicine Siriraj Hospital, Mahidol University, Bangkok 10700, Thailand

**Keywords:** nonalcoholic fatty liver disease, nonalcoholic steatohepatitis, vitamin D, *VDR*, *CYP27B1*, *CYP2R1*, *CYP24A1*, *GC*, *DHCR7*, genetic variation, polymorphism, systematic review

## Abstract

Background: Studies have demonstrated the link between vitamin-D-related genetic variations and nonskeletal outcomes. We aimed to identify all available data on the association of vitamin-D-related genetic variations with nonalcoholic fatty liver disease (NAFLD). Methods: Potentially eligible studies were identified from Embase and Medline databases from inception to June 2022 using a search strategy that comprised terms for “Vitamin D” and “NAFLD”. Eligible studies must report the association between vitamin D-related genetic variations and presence, severity or response to treatment of NAFLD. Data were extracted from each eligible study. Results: A total of 3495 articles were identified. After a systematic review, twelve studies were included. A total of 26 genetic variations were identified. Presence of NAFLD was associated with variations of *GC* (rs222054, rs222020, rs10011000, rs7041), *VDR* (rs2228570, rs11168287, rs10783219, rs4752), *CYP24A1* (rs3787557, rs6068816, rs2296241, rs2248359) and *CYP27B1* (rs4646536). Severity of NAFLD was associated with variations of *GC* (rs4588), *VDR* (rs2228570, rs4334089), *CYP2R1* (rs10741657), *DHCR7* (rs1544410, rs3829251, rs12785878) and *CYP24A1* (rs3787557, rs6068816, rs6097809, rs6127119, rs2248359, rs3787554, rs4809960, rs6022999). Response to calcitriol treatment was associated with variation of *VDR* (rs10735810). Conclusions: Multiple vitamin D-related genetic variations were associated with NAFLD, indicating the role of vitamin D in the pathogenesis of NAFLD.

## 1. Introduction

Nonalcoholic fatty liver disease (NAFLD) is defined by hepatic fat accumulation in the absence of any secondary causes such as excessive alcohol consumption and hepatic viral diseases [1]. It is a spectrum of diseases that range from fatty acid accumulation in the liver, inflammation of the liver causing nonalcoholic steatohepatitis (NASH), advanced fibrosis, cirrhosis and hepatocellular carcinoma. NAFLD is the most common chronic liver disease in the world and soon to be the most common indication for liver transplantation [1,2]. Previous studies reported a global prevalence of NAFLD as high as one billion individuals, and 25% of these patients progress to NASH [3,4]. NAFLD is known to be a common comorbidity in obesity and metabolic syndrome given its reported prevalence of 65–85% among patients with obesity [5].

Vitamin D is a steroid hormone responsible for regulating calcium and phosphate metabolism. It is also known to exert multitudes of nonskeletal effects given that vitamin D receptor (VDR) is expressed in various types of tissues and cells, including the skin, skeletal muscle, adipose tissue, endocrine pancreas and immune cells, among others [6,7]. Humans get vitamin D from diets (vitamin D_2_ from yeast and mushrooms and vitamin D_3_ from animal products) and endogenous synthesis in the skin (vitamin D_3_ from 7-dehydrocholesterol exposure to UVB). Once entering the circulation, vitamin D (D_2_ and D_3_) gets converted into 25-hydroxyvitamin D [25(OH)D], the major circulating form of vitamin D, by the hepatic enzyme 25-hydroxylase. Then, 25(OH)D gets metabolized by the enzyme 25-hydroxyvitamin D-1α-hydroxylase in the kidney into the active form 1,25(OH)_2_D, which interacts with the intracellular VDR in the target tissues. The 1,25(OH)2D-activated VDR then interacts with the retinoid X receptor (RXR), which selectively recognizes the vitamin D-responsive elements in the promotor sites of the target genes resulting in changes in gene expression [8]. 1,25(OH)_2_D and 25(OH)D both get catabolized by the enzyme 24-hydroxylase expressed in multiple tissues into inactive carboxylic acids, which are then excreted via the biliary system [6,7].

Genes associated with vitamin D metabolism include (1) *DHCR7*, encoding 7-dehydrocholesterol reductase, which metabolizes 7-dehydrocholesterol, a substrate of vitamin D [9]; (2) genes encoding the enzyme 25-hydroxylase, which converts vitamin D into the circulating form 25(OH)D, including *CYP2R1* (encoding the cytochrome P450 family 2 subfamily R member 1), *CYP2J2* (encoding the cytochrome P450 family 2 subfamily J polypeptide 2, known to hydroxylate vitamin D_2_ better than vitamin D_3_ [10]), *CYP27A1* (encoding the cytochrome P450 family 27 subfamily A member 1) and *CYP3A4* (encoding the cytochrome P450 family 3 subfamily A member 4), which converts vitamin D into the circulating form 25(OH)D [11]; (3) *CYP27B1*, encoding the chrome P450 family 27 subfamily B member 1, or the enzyme 25-hydroxyvitamin D-1α-hydroxylase, which converts 25(OH)D into the active form 1,25(OH)_2_D; (4) *GC*, encoding the *GC* vitamin D-binding protein; (5) *VDR*, encoding the vitamin D receptor; and (6) *CYP24A1*, encoding the cytochrome P450 family 24 subfamily A member 1, or the enzyme 24-hydroxylase, which catabolizes 25(OH)D and 1,25(OH)_2_D into inactive carboxylic acids [9].

Vitamin D deficiency, indicated by low level of serum 25(OH)D of less than 20 ng/mL [12,13] has been shown to be associated with multiple chronic diseases, including cardiovascular disease, diabetes, autoimmune diseases and cancers [6,7,14]. Low level of 25(OH)D is also found to be associated with presence and severity of NAFLD in multiple studies [15,16]. Several underlying mechanisms of the association have been proposed. These include the observation that vitamin D plays a role in modulating the immune system and improving insulin sensitivity [17,18]. However, the causality of this association is still unclarified given that there is limited evidence from clinical trials on the impact of vitamin D supplementation on NAFLD prevention and treatment [19].

Interestingly, variations of genes involved in the vitamin D metabolism pathway described above have been shown to be associated with many conditions, such as coronary artery disease, osteoporosis, diabetes and autoimmune diseases, indicating the interaction between this pathway and health outcomes [20,21,22,23]. Additionally, a number of studies have reported the association between vitamin D-related genetic variations and NAFLD [24,25,26,27,28,29,30,31,32,33,34,35]. The objective of this systematic review was therefore to identify and summarize the results of all available studies that explored the association of vitamin D-related genetic polymorphisms with presence, severity and response to treatment of NAFLD.

## 2. Methods

### 2.1. Search Strategy

Two investigators (A.J., N.C.) independently searched records indexed in Embase and Medline from inception to June 2022. The search strategy included terms related to nonalcoholic fatty liver disease, vitamin D and genes involved in the vitamin D metabolic pathway, as shown in Supplemental Material S1. The PRISMA guideline for systematic review was followed, as indicated in Supplemental Material S2. No language restriction was applied.

### 2.2. Eligibility Criteria

Eligible studies must be observational studies that investigated the association between presence, severity or response to treatment of NAFLD and genetic variations in vitamin D-related genes. These genes include *DHCR7*, *CYP2R1, CYP2J2*, *CYP27A1*, *CYP3A4*, *CYP27B1*, *GC*, *VDR* and *CYP24A1*.

Two investigators (A.J., B.P.) independently reviewed the titles and abstracts of retrieved records. Records that clearly did not fulfill the eligibility criteria based on type of article, study design or outcome of interest were excluded at this stage. Then, two investigators (A.J., B.P.) independently evaluated the full text of the remaining records for their final eligibility. The quality of each included study was assessed using the Newcastle–Ottawa quality assessment scale for the case–control study [36], which was performed by two investigators (AJ, BP). Different opinions in the eligibility and quality assessment of the records were resolved by discussion with the senior investigator (N.C.).

### 2.3. Data Extraction

Data from each eligible record were extracted using the standardized data collection form, which contained the following information: last name of the first author, country of the study, number of participants, evaluation of presence and/or severity of NAFLD, mean age of the participants, percentage of female participants and reported association of vitamin D-related genetic variations with outcomes.

## 3. Results and Discussion

### 3.1. Results

#### 3.1.1. Search Results

**Figure 1:**
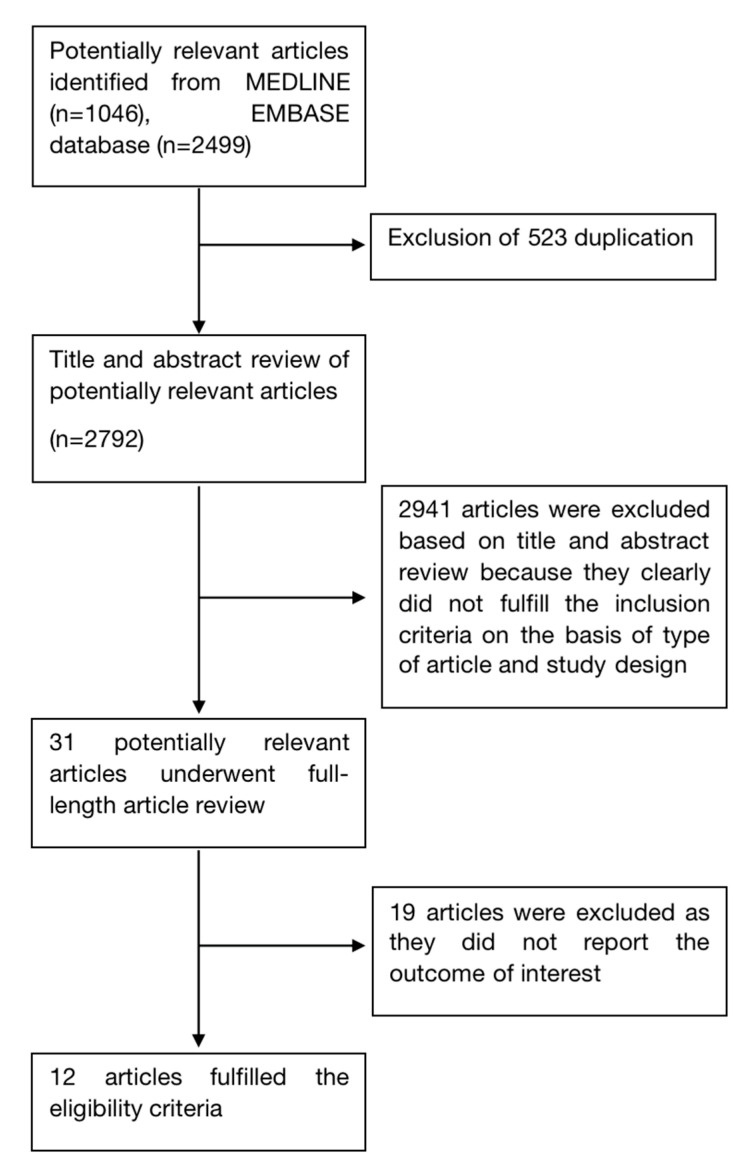
Study identification and literature review process.

A total of 3495 records were identified from the electronic search. After removal of 523 duplicates, 2972 records underwent title and abstract review. A total of 2941 records were excluded at this stage as they clearly did not fulfill the eligibility criteria based on type of article, study design and outcome of interest, leaving 31 records for full-text review. A total of 19 records were further excluded at this stage since they did not report the outcome of interest. Finally, a total of 12 studies fulfilled the eligibility criteria [24,25,26,27,28,29,30,31,32,33,34,35]. Figure 1 summarizes the literature search and review process of this study.

#### 3.1.2. Characteristics of Studies Reporting the Association between Vitamin D-Related Genetic Variations and Presence and/or Severity of NAFLD

A total of 12 studies consisting of at least 18,012 participants combined reported the outcome of interest [24,25,26,27,28,29,30,31,32,33,34,35]. These studies were conducted from 2010 to 2022. Four studies (two from the same population) are from China [30,31,32,35], three studies from the same group are from the United Kingdom [26,27,28], and the rest are from Australia [24], Germany, Iran [33], Japan [25] and the United States [34]. The average age of participants varied from 13.8 to 55.0 years and the percentage of females varied from 16.7 to 60.0%. Five studies investigated the association between vitamin D-related genetic variations and presence of NAFLD [24,26,30,34,35], six studies explored the relationship between the genetic variations and severity of NAFLD [26,27,28,29,34] and one study explored the influence of *VDR* genetic variation on response to calcitriol treatment [33]. Evaluations of NAFLD were performed by liver ultrasound in five studies [24,31,32,33,35], liver biopsy in four studies [25,26,27,28] and abdominal computed tomography in one study [34]. Eight of the twelve studies were of high quality based on a Newcastle–Ottawa score of more than 7 [24,25,29,30,31,32,34,35]. Five studies performed multivariate analysis to adjust for potential confounders [25,29,30,31,35]. The characteristics of all included studies were summarized in Table 1.

#### 3.1.3. Association between Vitamin D-Related Polymorphism and Presence, Severity and Response to Treatment of Nonalcoholic Fatty Liver Disease

Among the 12 included studies, a total of 26 genetic variations of six genes were identified to be associated with presence, severity or response to treatment of NAFLD, including *GC*, *VDR*, *CYP27B1*, *CYP2R1*, *DHCR7* and *CYP24A1*. As shown in Table 2, the presence of NAFLD was associated with variations of the genes *GC* (rs222054, rs222020, rs10011000, rs7041), *VDR* (rs2228570, rs11168287, rs10783219, rs4752), *CYP24A1* (rs3787557, rs6068816, rs2296241, rs2248359) and *CYP27B1* (rs4646536) [24,26,30,31,32,34]. Liver density based on abdominal computed tomography was associated with variations of the genes *VDR* (rs4334089) and *CYP24A1* (rs3787557, rs6068816, rs6097809, rs6127119, rs2248359, rs3787554, rs4809960, rs6022999) [34]. Histological steatosis was associated with variations of the genes *DHCR7* (rs3829251) and *VDR* (rs2228570) [28,29]. In addition, another *DHCR7* variation (rs12785878) was found to be associated with histological steatosis in one study [28] but not in the other [29]. Inflammation and fibrosis based on NAFLD activity score were associated with mutations in the genes *DHCR7* (rs12785878), *GC* (rs4588), *VDR* (rs2228570) and *CYP2R1* (rs10741657) [28]. Advanced fibrosis based on NAFLD activity score was associated with *VDR* (rs1544410) genetic variation [25]. Overall NAFLD activity score was associated with *CYP2R1* (rs10741657) [27]. Finally, response to calcitriol treatment indicated by the degree of decrease in alkaline phosphatase was associated with *VDR* (rs10735810) genetic variation [33].

### 3.2. Discussion

This is the first systematic review that explored the relationship between vitamin D-related genetic variations and NAFLD. Our systematic review revealed the association of presence, severity and response to treatment of NAFLD with variations of several genes in the vitamin D metabolic pathway, which include *DHCR7*, *CYP2R1*, *CYP24A1*, *CYP27B1*, *GC* and *VDR*. These findings support the notion that the vitamin D signaling pathway may play a significant role in the pathogenesis of NAFLD.

In fact, 1,25(OH)_2_D, the active form of vitamin D is known to have anti-inflammatory and antifibrogenic effects by inhibiting proinflammatory cytokines production (i.e., interleukin-1, interleukin-6 and tumor necrosis factor-alpha), enhancing anti-inflammatory cytokines production (i.e., interleukin-10 and adiponectin) and suppressing the function hepatic stellate cells [17,37,38]. These actions could therefore slow down the process of liver inflammation and fibrosis. In addition, 1,25(OH)_2_D was shown to enhance insulin receptor expression, thereby mitigating insulin resistance, which is known to be a major component of NAFLD pathogenesis [39,40].

It is worth noting that multiple observational studies revealed the association between low level of serum 25(OH)D and increased risk and severity of NAFLD [15]. Nevertheless, the causality and clinical significance of this association remain undetermined given that the association could be due to confounding effects (i.e., limited physical activity and high body mass index) as well as reverse causation since hepatic steatosis is shown to be associated with decreased activity of the 25-hydroxylase, which can result in decreased circulating 25(OH)D [15,16]. A few randomized controlled trials have shown that vitamin D supplementation can improve hepatic steatosis and insulin resistance and decrease biomarkers of inflammation in adults with NAFLD [41]. However, a mendelian randomization study by Wang et al. demonstrated no causal association between serum 25(OH)D concentration and presence of NAFLD [32].

Alteration of any steps of vitamin D metabolism could possibly affect levels and functions of vitamin D. For example, previous genome-wide association studies reported variations in the *DHCR7*(rs12785878), *GC* (rs2282679), *CYP2R1*(rs10741657), *VDR* (rs2228570, rs1544410, rs7975232, rs731236) and *CYP24A1* (rs17216707) genes were associated with serum 25(OH)D level [42,43]. Another meta-analysis found that genetic variations of the *GC* (rs2282679, rs4588, rs1155563, rs7041) and *CYP2R1* genes (rs10741657, rs10766197, rs2060793) were associated with vitamin D levels in more than 50% of the respective studies [44]. Therefore, variations in genes related to vitamin D metabolism may eventually affect the risks of NAFLD from multiple possible pathophysiologies mentioned above. It has been shown in an animal model that mice lacking *VDR* in the liver had increased hepatic steatosis and insulin resistance, as well as diminished the protective effect of vitamin D supplementation on NAFLD [45]. Mechanistic experiments demonstrated that the *VDR* interaction may improve lipid metabolism by interacting with the hepatocyte nuclear factor 4 α (HNF4α) [45]. Based on this observation, it is possible that genetic variations in the *VDR* may affect *VDR* expression in the liver, thereby modifying the risk of NAFLD. Data on animal models associated with other genes in the vitamin D metabolic pathway are, however, lacking.

Taken together, the relationship between vitamin D and pathogenesis and progression of NAFLD remains unclarified and may be complex. The observation that genetic variations of vitamin D-related genes were associated with presence, severity and response to treatment of NAFLD provides additional insights, as it may indicate interindividual differences in responsiveness to vitamin D. This can be supported by the observation by Barchetta et al. [46] in patients with NASH that vitamin D receptor expression on cholangiocytes was negatively correlated with steatosis severity, lobular inflammation and NAFLD score. Furthermore, the concept of individual responsiveness to vitamin D has been introduced based on findings from clinical trials that revealed differences in the degree of genome-wide expression in peripheral blood mononuclear cells as well as metabolomic profiles in response to vitamin D supplementation [47,48,49]. Future studies are warranted to further explain the impact of vitamin D-related genetic variations on metabolic outcomes.

The results of this systematic review may have some research implications as the reported genetic variations associated with NAFLD may be used as a reference for future animal models to further elucidate the link between vitamin D and NAFLD pathogenesis. In addition, these variations may be considered novel markers for determining individuals at risk for developing NAFLD. However, there are certain limitations of this systematic review that should be acknowledged. Most of the included studies are small in sample size, and many of them did not adjust for confounders as only five studies were performed in a large-scale cohort [24,31,32,34,35], and four studies conducted robust multivariate analysis [25,30,31,35]. Notably, none of the included studies considered calcium supplementation, a potential confounder that could affect NAFLD pathogenesis, in their multivariate analyses [50]. More importantly, none of the reported significant genetic variations were confirmed to be associated with outcomes in more than one study. All these could have jeopardized the reliability of the findings. Further studies are required to verify the findings of these studies.

## 4. Conclusions

This systematic review identified 26 vitamin D-related genetic variations in the *DHCR7*, *CYP2R1*, *CYP24A1*, *CYP27B1*, *GC*, and *VDR* genes to be associated with presence, severity or response to treatment of NAFLD. However, the confidence of these findings was relatively limited awaiting confirmation by future studies.

## Figures and Tables

**Table 1:** Main characteristics of studies investigating the association between vitamin D-related genetic variations and nonalcoholic fatty liver disease included in the systematic review.

Study	Young [34]	Adams [24]	Gibson [27]
Country	USA	Australia	UK
Year of publication	2010	2012	2014
Total number of participants	1180	928	78
Recruitment of participants	Cases were patients with NAFLD recruited from families in San Antonio, San Luis Valley, and Los Angeles, USA who had CT measured of liver and visceral fat Controls were patients without NAFLD recruited from the same sites during the same period	Cases were patients with NAFLD recruited from the Western Australian Pregnancy Cohort (Raine) Study in Perth, Western Australia from 1989 to 1992 Controls were patients without NAFLD recruited from the same sources during the same period	Cases were NAFLD patients with increased fibrosis of liver (F2–F4) or increased NAS score recruited from medical records from the King’s College Hospital Pediatric Liver Clinic, UK Controls were NAFLD patients without increased fibrosis of liver (F2–F4) or increased NAS score recruited from the same source during the same period
Evaluation of NAFLD	CT measure of the liver and visceral fat	Liver ultrasound at age 17	Liver biopsy
Average age of participants (years)	48.7	Cases: 17.0 Controls: 17.0	N/A
Percentage of female participants	62.4	Cases: 60.3 Controls: 45.9	N/A
Variables adjusted in multivariate analysis	N/A	N/A	N/A
Newcastle–Ottawa score	Selection: 4 Comparability: 0 Exposure: 3	Selection: 4 Comparability: 0 Exposure: 3	Selection: 3 Comparability: 0 Exposure: 3
Study	Gibson [26]	Gibson [28]	Jamka [29]
Country	UK	UK	Germany
Year of publication	2015	2018	2018
Total number of participants	103	103	241
Recruitment of participants	Participants were patients with NAFLD recruited from medical records from the King’s College Hospital Pediatric Liver Clinic, UK, from March 2001 to July 2013	Participants were patients with NAFLD recruited from medical records from the King’s College Hospital Pediatric Liver Clinic, UK, from March 2001 to July 2013	Participants were patients with chronic liver diseases recruited from the Department of Medicine II, Saarland University Medical Center, Homburg, Germany
Evaluation of NAFLD	Liver biopsy	Liver biopsy	Fibroscan
Average age of participants (years)	13.8	13.8	55.0
Percentage of female participants	34.0	34.0	43.1
Variables adjusted in multivariate analysis	N/A	N/A	Age, season
Newcastle–Ottawa score	Selection: 3 Comparability: 0 Exposure: 3	Selection: 3 Comparability: 0 Exposure: 3	Selection: 3 Comparability: 1 Exposure: 3
Study	Wang [32]	Arai [25]	Wang [31]
Country	China	Japan	China
Year of publication	2018	2019	2021
Total number of participants	9182	229	3025
Recruitment of participants	Cases were patients with NAFLD recruited from adult Chinese citizens from 23 sites in Shanghai, Zhejiang, Jiangsu, Anhui, and Jiangxi provinces from 2014 to 2016 Controls were patients without NAFLD recruited from the same sites during the same period	Cases were NAFLD patients with advanced fibrosis of liver (F3–F4) recruited from families in San Antonio, San Luis Valley, and Los Angeles Controls were NAFLD patients without advanced fibrosis of liver (F3–F4) recruited from the same sites during the same period	Cases were patients with NAFLD recruited from a community in Nanjing, Jiangsu, China from July to September 2018 Controls were patients without NAFLD recruited from the same source during the same period
Evaluation of NAFLD	Liver ultrasound	Liver biopsy	Liver ultrasound
Average age of participants (years)	54.0	55.0	Cases: 40.4 Controls: 39.7
Percentage of female participants	64.0	46.7	Cases: 15.5 Controls: 18.1
Variables adjusted in multivariate analysis	N/A	Age, body mass index, total cholesterol, serum 25(OH)D_3_	Age, sex, visceral obesity, ALT, GGT, hypertension, hypertriglyceridemia, hyperglycemia, low HDL-C, unfavorable alleles
Newcastle–Ottawa score	Selection: 4 Comparability: 0 Exposure: 3	Selection: 3 Comparability: 2 Exposure: 3	Selection: 3 Comparability: 2 Exposure: 3
Study	Yaghooti [33]	Zhang [35]	Wang [30]
Country	Iran	China	China
Year of publication	2021	2021	2022
Total number of participants	128	3023	N/A
Recruitment of participants	Participants were patients with fatty liver recruited from patients referred to the Ahvaz Golestan Hospital, Iran, from 2017 to 2018	Cases were patients with NAFLD recruited from a community in Nanjing, Jiangsu, China, from July to September 2018 Controls were patients without NAFLD recruited from the same source during the same period	Cases were patients with NAFLD recruited from Chinese population Controls were patients without NAFLD recruited from the same source during the same period
Evaluation of NAFLD	Liver ultrasound	Liver ultrasound	N/A
Average age of participants (years)	N/A	Cases: 40.5 Controls: 39.9	N/A
Percentage of female participants	N/A	Cases: 15.4 Controls: 17.5	N/A
Variables adjusted in multivariate analysis	N/A	Age, visceral obesity, hypertension, hypertriglyceridemia, low HDL-C, ALT, exercise	Age, gender, overweight, abdominal obesity, hypertension, hypertriglyceridemia, hyperglycemia
Newcastle–Ottawa score	Selection: 3 Comparability: 0 Exposure: 3	Selection: 4 Comparability: 2 Exposure: 3	Selection: 3 Comparability: 2 Exposure: 3

Abbreviations: 25(OH)D_3_: 25-hydroxyvitamin D_3_; ALT: Alanine aminotransferase; CT: Computed tomography; GGT: Gamma-glutamyl transferase; HDL-C: High-density lipoprotein-cholesterol; N/A: Not available; NAFLD: Nonalcoholic fatty liver disease; NAS: Nonalcoholic fatty liver disease activity score; UK: United Kingdom; USA: United States of America.

**Table 2:** Association between vitamin D-related genetic variations and presence, severity and response to treatment of nonalcoholic.

Outcome	Gene	Locus	Finding
Presence of NAFLD	*GC*	rs222054	- G allele association with 2.54-fold increased odds of NAFLD compared with C allele (Adams et al., 2012 [24])
	*GC*	rs222020	- C allele associated with 1.89-fold increased odds of NAFLD in AA (Young et al., 2010 [34])
	*GC*	rs10011000	- G allele associated with 1.96-fold increased odds of NAFLD in AA (Young et al., 2010 [34])
	*GC*	rs7041	- G allele associated with 0.81-fold decreased odds of NAFLD compared with T allele (Wang et al., 2022 [30])
	*GC*	rs2282679	- No association (Gibson et al., 2015 [26], Wang et al., 2018 [32], Wang et al., 2022 [30])
	*GC*	rs222020	- No association (Wang et al., 2022 [30])
	*GC*	rs4588	- No association (Wang et al., 2022 [30])
	*GC*	rs1155563	- No association (Wang et al., 2022 [30])
	*GC*	rs16847024	- No association (Wang et al., 2022 [30])
	*GC*	rs3733359	- No association (Wang et al., 2022 [30])
	*VDR*	rs2228570	- AA variant associated with 0.78-fold decreased odds of NAFLD compared with CC variant (Zhang et al., 2021 [35])
	*VDR*	rs11168287	- GA variant associated with 0.83-fold decreased odds of NAFLD compared with GG variant (Zhang et al., 2021 [35])
	*VDR*	rs10783219	- A allele associated with 3.7-fold increased odds of NAFLD in AA (Young et al., 2010 [34])
	*VDR*	rs4752	- T allele associated with 3.09-fold increased odds of NAFLD in AA (Young et al., 2010 [34])
	*CYP24A1*	rs3787557	- C allele associated with 2.60-fold increased odds of NAFLD in AA (Young et al., 2010 [34])
	*CYP24A1*	rs6068816	- C allele associated with 1.59-fold increased odds of NAFLD in HA (Young et al., 2010 [34])
	*CYP24A1*	rs2296241	- AA variant associated with 1.34-fold increased odds of NAFLD compared with GG variant (Wang et al., 2021 [31])
	*CYP24A1*	rs2248359	- TT variant associated with 1.35-fold increased odds of NAFLD compared with CC variant (Wang et al., 2021 [31])
	*CYP24A1*	rs6013897	- No association (Wang et al., 2018 [32])
	*CYP27B1*	rs4646536	- TT variant associated with 1.36-fold increased odds of NAFLD compared with CC variant (Wang et al., 2021 [31])
	*CYP2R1*	rs10741657	- No association (Gibson et al., 2015 [26], Wang et al., 2018 [32])
	*DHCR7*	rs12785878	- No association (Wang et al., 2018 [32])
Liver density ^a^	*VDR*	rs4334089	- Significant association in HA (Young et al., 2010 [34])
	*CYP24A1*	rs3787555	- Significant association in HA (Young et al., 2010 [34])
	*CYP24A1*	rs6068816	- Significant association in HA (Young et al., 2010 [34])
	*CYP24A1*	rs6097809	- Significant association in HA (Young et al., 2010 [34])
	*CYP24A1*	rs6127119	- Significant association in HA (Young et al., 2010 [34])
	*CYP24A1*	rs2248359	- Significant association in AA (Young et al., 2010 [34])
	*CYP24A1*	rs3787554	- Significant association in AA (Young et al., 2010 [34])
	*CYP24A1*	rs4809960	- Significant association in AA (Young et al., 2010 [34])
	*CYP24A1*	rs6022999	- Significant association in AA (Young et al., 2010 [34])
	*CYP27B1*	N/A	- No association (Young et al., 2010 [34])
	*CYP2R1*	N/A	- No association (Young et al., 2010 [34])
Steatosis ^b^	*DHCR7*	rs12785878	- Significant association (Gibson et al., 2018 [28]) - No association (Jamka et al., 2018 [29])
	*DHCR7*	rs3829251	- Significant association (Gibson et al., 2018 [28])
	*GC*	rs7041	- No association (Jamka et al., 2018 [29])
	*VDR*	rs2228570	- Significant association (Gibson et al., 2018 [28])
	*VDR*	rs79754353	- No association (Jamka et al., 2018 [29])
	*CYP2R1*	rs10741657	- No association (Jamka et al., 2018 [29])
Inflammation and fibrosis ^c^	*DHCR7*	rs12785878	- G allele associated with NAFLD steatosis, inflammation and fibrosis (Gibson et al., 2018 [28])
	*GC*	rs4588	- Significant association (Gibson et al., 2018 [28])
	*VDR*	rs2228570	- Significant association (Gibson et al., 2018 [28])
	*CYP2R1*	rs10741657	- Significant association (Gibson et al., 2018 [28])
Advanced fibrosis ^d^	*DHCR7*	rs7944926	- No association (Arai et al., 2019 [25])
	*DHCR7*	rs12785878	- No association (Arai et al., 2019 [25])
	*GC*	rs2282679	- No association (Arai et al., 2019 [25])
	*VDR*	rs1544410	- Variant CC associated with 4.04-fold increased odds of advanced fibrosis compared with non-CC (Arai et al., 2019 [25])
	*VDR*	rs2228750	- No association (Arai et al., 2019 [25])
	*VDR*	rs7975232	- No association (Arai et al., 2019 [25])
	*VDR*	rs731236	- No association (Arai et al., 2019 [25])
	*CYP27B1*	rs10877012	- No association (Arai et al., 2019 [25])
	*CYP2R1*	rs1993116	- No association (Arai et al., 2019 [25])
	*CYP2R1*	rs10741657	- No association (Arai et al., 2019 [25])
NAFLD activity score ^e^	*CYP2R1*	rs10741657	- G allele associated with increased NAFLD activity score (Gibson et al., 2014 [27])
NAFLD histological severity ^f^	*GC*	rs2282679	- No association (Gibson et al., 2015 [26])
	*CYP2R1*	rs10741657	- No association (Gibson et al., 2015 [26])
Response to calcitriol treatment	*VDR*	rs10735810	- Ff genotype associated with higher decrease in ALP activity in NAFLD patient in response to calcitriol treatment compared with FF genotype (Yaghooti et al., 2021 [33])

^a^ Liver density was examined using a variance components approach based on computed tomography measure of the liver and visceral fat. ^b^ Steatosis was assessed based on histology using the NAFLD activity score (Gibson et al., 2018 [28]) and controlled attenuation parameter from transient elastography (Jamka et al., 2018 [29]). ^c^ Inflammation and fibrosis were assessed using the NAFLD activity score (Gibson et al., 2018 [28]). ^d^ Histopathological evaluation was performed by experienced pathologists blinded to clinical and laboratory data of the patients. Liver fibrosis was semi-quantitatively evaluated using the NASH Clinical Research Network scoring system. ^e^ The NAFLD activity score included a summation of numerical scores for steatosis (0–3), hepatocyte ballooning (1–2) and lobular inflammation (0–3) (Gibson et al., 2018 [28]). ^f^ Biopsies were scored by a liver histopathologist according to the Kleiner–Brunt system. Abbreviations: AA: African American; ALP: Alkaline phosphatase; HA: Hispanic American; N/A: Not applicable; NAFLD: Nonalcoholic fatty liver disease; NASH: Nonalcoholic steatohepatitis.

## Supplementary Materials

The following supporting information can be downloaded at: https://www.mdpi.com/article/10.3390/ijms23169122/s1. Ref. [51] is cited in Supplementary Materials.
